# Differential Post-Translational Modifications of Proteins in Bladder Ischemia

**DOI:** 10.3390/biomedicines12010081

**Published:** 2023-12-28

**Authors:** Han-Pil Choi, Jing-Hua Yang, Kazem M. Azadzoi

**Affiliations:** 1 Proteomics Laboratory, VA Boston Healthcare System, Boston, MA 02130, USA; han-pil.choi@va.gov; 2 Proteomics Laboratory, Department of Surgery, VA Boston Healthcare System, Boston University School of Medicine, Boston, MA 02130, USA; jyangva@gmail.com; 3 Departments of Urology and Pathology, VA Boston Healthcare System, Boston University School of Medicine, Boston, MA 02130, USA

**Keywords:** bladder, ischemia, protein profiling, post-translational modifications, non-coded amino acids

## Abstract

Clinical and basic research suggests that bladder ischemia may be an independent variable in the development of lower urinary tract symptoms (LUTS). We have reported that ischemic changes in the bladder involve differential expression and post-translational modifications (PTMs) of the protein’s functional domains. In the present study, we performed in-depth analysis of a previously reported proteomic dataset to further characterize proteins PTMs in bladder ischemia. Our proteomic analysis of proteins in bladder ischemia detected differential formation of non-coded amino acids (ncAAs) that might have resulted from PTMs. In-depth analysis revealed that three groups of proteins in the bladder proteome, including contractile proteins and their associated proteins, stress response proteins, and cell signaling-related proteins, are conspicuously impacted by ischemia. Differential PTMs of proteins by ischemia seemed to affect important signaling pathways in the bladder and provoke critical changes in the post-translational structural integrity of the stress response, contractile, and cell signaling-related proteins. Our data suggest that differential PTMs of proteins may play a role in the development of cellular stress, sensitization of smooth muscle cells to contractile stimuli, and deferential cell signaling in bladder ischemia. These observations may provide the foundation for future research to validate and define clinical translation of the modified biomarkers for precise diagnosis of bladder dysfunction and the development of new therapeutic targets against LUTS.

## 1. Introduction

Proteins are produced in different shapes, sizes, and forms. Proteins play a fundamental role in the regulation of major processes of cell function and dysfunction, with an enigmatic position between human genetics and the varying phenotypes of both health and disease [1]. Systematic studies quantifying transcripts and proteins have revealed that transcript levels do not necessarily predict protein levels in many circumstances [2]. The spatial and temporal variations in the availability of precursors for protein biosynthesis and post-translational conformational changes in protein structure strongly influence their expression patterns and bioavailability [3]. There are multiple processes beyond the transcript level that modulate the expression level of a protein, including translation rates, modulation of protein half-life, protein synthesis delay, protein transport, and post-translational modifications (PTMs) [4]. Many proteins undergo PTMs that could immensely influence their structural integrity and function. PTMs of proteins activate important signaling pathways and regulate many biological processes [1,2,3,4]. Analyzing the diversity of PTMs is crucial for understanding downstream mechanisms of cell regulation. Mass spectrometry-based proteomic technologies are fundamental tools for identifying and mapping covalent modifications, providing an exceptional opportunity to detect and analyze a large number of differentially expressed protein variants in disease conditions [5]. Modern proteomic approaches have made large-scale assessments possible, enabling the mapping of modification sites and the quantification of the modified sites of proteins.

Despite tremendous advances in our understanding of the pathophysiology of systemic disorders, there are still substantial gaps in defining the precise molecular basis for early diagnosis and effective treatment of most disease conditions. Further knowledge toward a better understanding of differentially expressed proteins and protein modifications in tissues, cells, subcellular elements, and biological fluids may help the development of novel biomarkers for early diagnosis of systemic disorders and could lead to new therapeutic targets with the potential of new drug development through novel strategies. Proteomic approaches in these areas involve the comparative assessment of protein expression and modifications in disease conditions to detect aberrantly expressed proteins with the potential of illuminating new biomarkers. Thus, mass spectrometry-based proteomic studies of altered and modified proteins may have great potential to yield comprehensive profiles of proteins in tissues and cells for the potential identification of diagnostic and therapeutic markers.

Lower urinary tract symptoms (LUTS) increase with aging in both men and women [6,7,8,9]. Prostatic enlargement and bladder outlet obstruction (BOO) are the leading causes of LUTS in men. However, growing evidence suggests that all cases of LUTS in men are not necessarily due to prostatic enlargement or BOO, suggesting other aging-associated causes [10,11,12,13,14]. It is widely accepted that aging-associated LUTS involve multiple possible etiologies, including primary detrusor overactivity, compromised bladder smooth muscle contractility, or a combination of the two. A possible mechanism of non-obstructed bladder dysfunction and LUTS receiving increasing attention is the involvement of pelvic arterial atherosclerosis and bladder exposure to ischemia [6,7,8,9,10,11,12,13,14,15,16,17,18,19,20,21,22,23,24,25,26]. Ischemia was shown to interfere with lower urinary tract function and is believed to be an independent variable in the development of non-obstructed non-neurogenic detrusor overactivity and LUTS [6,7,8,9,10,11,12,13,14,15,16,17,18,19,20,21,22,23,24,25,26]. Ischemia alters the proteomic profiles of many organs, including the heart [27], brain [28,29], and intestine [30], but its impact on the bladder proteome remains largely elusive. Recent proteomic profiling using liquid chromatography tandem mass spectrometry (LC-MS/MS) has revealed critical information on alterations and modifications of proteins in bladder ischemia [31,32]. Differential expression and post-translational modifications (PTMs) of proteins appear to play important roles in ischemia-provoked bladder dysfunction [31,32]. We have previously reported that a total of 359 proteins are differentially expressed in bladder ischemia with twofold changes [32]. Gene ontology analysis suggested that differentially expressed proteins with fivefold changes are involved in molecular mechanisms underlying proteolysis and degenerative processes [32]. In the bladder proteome, a large number of nonzero delta masses (11,056) were detected, spanning over 1295 protein residues and grouped into 23 delta mass clusters, where each cluster represented a specific chemical reaction occurring on the side chain of the modified proteins. Among them, 12 delta mass clusters were significantly dysregulated in bladder ischemia (R^2^ > 0.5, ratio > twofold, *p* < 0.05), which were considered potential ischemia-regulated protein modifications [31]. Pathway analysis suggested the involvement of altered proteins in heat shock transcription factor 1-dependent transactivation, smooth muscle contraction, attenuation phase, and tetrahydrobiopterin synthesis, recycling, salvage, and regulation pathways [31]. These observations suggest that ischemia initiates a series of complex molecular responses through differential protein expression and massive PTMs in the bladder. It was shown that alterations of the bladder proteome by ischemia trigger a cascade of downstream signaling pathways with critical structural and functional consequences [31,32]. Differential expression and PTMs of proteins in bladder ischemia were associated with increased contractile activity consistent with detrusor overactivity, loss of smooth muscle cells, diffuse fibrosis, non-compliance, neural structural damage, and marked changes in the ultrastructure of the subcellular elements [31,32]. In this study, we focused on the proteomic assessment of bladder ischemia to define comprehensive differential (post-translational) modifications of the stress response, contractile, and cell signaling-related proteins and determine activation of downstream pathways in an animal model.

## 2. Materials and Methods

### 2.1. Bladder Ischemia Model

Animal care and experimental procedures followed protocol number 396-J-0522 and guidelines approved by the Institutional Animal Care and Use Committee (IACUC) at VA Boston Healthcare System. Our IACUC is accredited by the Association for Assessment and Accreditation of Laboratory Animal Care (AAALAC). A total of six male Sprague-Dawley rats, 10–12 weeks old, weighing 200–400 g, were used. Control rats were obtained from Charles River Laboratories in Boston, Massachusetts, USA. Bladder ischemia was produced in apolipoprotein E knockout rats using balloon endothelial denudation of the iliac arteries with a 2F Fogarty arterial embolectomy catheter to produce atherosclerotic occlusive disease, as we have previously reported [31,32,33]. Sham control rats underwent similar surgical procedures without arterial endothelial denudation. After 8 weeks, bladder ischemia was verified by measuring microcirculatory bladder perfusion using the Moor FLPI-2 Laser Speckle Contrast Imaging system (Moor Instruments, Wilmington, DE, USA). This system provides blood flow imaging in a real-time and contact-free manner and is capable of measuring tissue microcirculation blood flow up to a tissue depth of 3 mm. To obtain blood flow measurements, the animals were anesthetized, and then a lower abdominal incision was made to expose the bladder. The speckle laser was positioned 25 cm above the bladder, and blood perfusion was recorded for 30 s at five different sites of the bladder. Mean bladder blood perfusion was analyzed using the moorFLPI-2 Review Software, v. 4.0 (Moor Instruments, Wilmington, DE, USA). After blood flow measurement, the animals were euthanized, and bladder tissues were prepared and processed for liquid chromatography-tandem mass spectrometry (LC-MS/MS) proteomic analysis as described below.

### 2.2. Sample Preparation for Proteomic Analysis 

Bladder tissue samples were collected from three animals with bladder ischemia and three sham control bladders and processed for proteomic analysis. Bladder tissue samples were homogenized in 1 mL of 1X RIPA lysis buffer (Millipore 20–188, MilliporeSigma, Burlington, MA, USA) supplemented with 10 µL of 500 mM DTT and 10 µL of protease inhibitor cocktail (MilliporeSigma, Burlington, MA, USA), and sonicated three times for 5 s on ice. The tissue lysates were centrifuged, the supernatants were collected, and protein concentrations were determined with the bicinchoninic acid (BCA) assay (MilliporeSigma, Burlington, MA, USA).

### 2.3. Sodium Dodecyl Sulfate Polyacrylamide Gel Electrophoresis (SDS-PAGE) Fractionation and Trypsin Digestion

The proteins were fractionated using one-dimensional SDS-PAGE. One hundred micrograms of each bladder tissue lysate protein were run into each well on the SDS-PAGE gel. The gel was then stained with Coomassie Brilliant Blue, and each gel lane was subsequently divided into 10 fractions based on molecular weight. Gel slices underwent the following steps: washing with 50 mM ammonium bicarbonate, dehydration with acetonitrile, and lyophilization in a SpeedVac (Thermo Scientific, Waltham, MA, USA). Subsequently, proteins were in-gel digested with sequencing-grade modified trypsin (Promega, Madison, WI, USA) at a protein-to-trypsin ratio of 25:1 in 50 mM ammonium bicarbonate (pH 8) overnight at 37 °C. The extracted peptides from each gel slice were subjected to desalting using ZipTip C18 columns (MilliporeSigma, Burlington, MA, USA) and then dried in a SpeedVac before LC-MS/MS analysis. The raw data for the 10 fractions from each tissue sample were searched against the UniProt database as a group.

### 2.4. Liquid Chromatography-Tandem Mass Spectrometry (LC-MS/MS) Analysis

The goal was to explore proteomic modifications in bladder ischemia. Therefore, the calculated statistical differences described in this report are not hypothetical. The desalted peptides were subjected to peptide fractionation using an EASY-nLC 1000 system (Thermo Scientific, Waltham, MA, USA). Samples were initially trapped on a C18 pre-column and subsequently fractionated using a long C18 column (300 × Ø 0.075 mm, 3 μm; Reprosil, Germany) employing a 180-min linear gradient of 5–35% acetonitrile/0.1% formic acid at a flow rate of 250 nL/min. Solvent A consisted of 0.1% formic acid in water, while solvent B comprised 0.1% formic acid in acetonitrile. MS and MS/MS spectra were acquired using an LTQ-Orbitrap Elite mass spectrometer (Thermo Scientific, Waltham, MA, USA) in a data-dependent mode. Each biological sample was replicated three times in the LC-MS/MS analysis, and the delta masses were individually subjected to an open search. Subsequently, the delta masses from all samples were clustered, and the average of the three replicates was given. The spray voltage was set at 2.1 kV, and the capillary temperature was maintained at 275 °C. MS spectra were acquired in profile mode within the *m*/*z* range of 350 to 1800 at a resolution of 60,000 at 400 *m*/*z*. The automatic gain control was set at 1 × 10^6^. MS/MS fragmentation was applied to the 30 most intense peaks for every full MS scan in the collision-induced dissociation mode. Normalized collision energies were fixed at 35%, with an activation time of 10 ms for the MS/MS method. The maximum precursor ion injection time for MS/MS was set at 100 ms. A repeat count of 2 and a dynamic exclusion duration of 90 s were applied. The minimal signal required for MS/MS was set at 1000.

### 2.5. The Clustered Delta Masses 

Typically, MS/MS spectra undergo a database search against the rat protein database using wildcard search functionality within the Byonic software (Version 3.6, Protein Metrics Inc., Cupertino, CA, USA) [34]. Results were then filtered based on criteria including Score ≥ 300, DeltaModsScore ≥ 10, FDR2D ≤ 0.01, and FDR_uniq.2D ≤ 0.01. Amino acid residues differing from coded genomic sequences were identified to calculate nonzero delta masses (delta mass > 0.01), representing the mass disparities between actual and theoretical (coded) amino acids. These amino acids with nonzero delta masses were referred to as “non-coded amino acids (ncAAs)”. For occurrence analysis, delta masses were rounded to four decimal places, and the number of redundancies was considered as the frequency of the delta mass. In clustering, delta masses were grouped into subcategories with 1-Da intervals, defined by *n* − 0.5 and *n* + 0.5 Da (where *n* ranged from −200 to 1000). The delta masses within each mass window were subjected to multivariate clustering using Gaussian mixture components [35]. Specific constraints were applied, including (1) a peak half-width > 1 ppm, (2) a peak distance > 2 peak widths, and (3) a cluster size > 20. The determination of clusters within each window was based on the Bayesian information criterion (BIC) [36], where larger BIC values indicated a stronger model and greater confidence in the number of clusters. Clusters within each window were individually fitted with Gaussian regression to calculate parameters such as the peak value (clustered delta mass), standard deviation (SD), and goodness-of-fit (R^2^). Clustered delta masses were annotated with known PTMs and amino acid substitutions found in databases like UniMod, RESID, ExPASy, and ABRF. Matched clusters were regarded as true delta masses present in the analyzed protein samples. Unmatched clusters were assigned with confidence only when the goodness-of-fit R^2^ > 0.5 and/or when the cluster was predominantly associated with a single amino acid (>50%). The significance of delta masses (modifications) was determined using parameters from a Gaussian distribution, assuming random errors. Thus, a significant delta mass was defined with R^2^ > 0.5, which represents a very high standard for non-linear regression. When comparing 3 ischemia samples to 3 controls, only ratios > 2-fold with *p* < 0.05 between the ischemia and control group were considered “significant”. Top of Form.

### 2.6. Statistical Analysis

Statistical analysis was performed using IBM SPSS 19.0 software (Armonk, NY, USA). Group comparisons were conducted using two-tailed Student’s *t*-tests, and an ANOVA with Tukey’s Honest Significant Difference (HSD) post-hoc test was utilized to assess variation between the groups. Significant differences were determined at the *p* < 0.05 level. *p*-values in proteomic data are based on a comparison of samples from three rats with bladder ischemia versus samples from three control rats. The term “significant” refers to changes in the bladder ischemia group in comparison with controls with a *p* value of <0.05. 

## 3. Results

### 3.1. Validation of Bladder Ischemia in the Rat Model 

**Figure 1:**
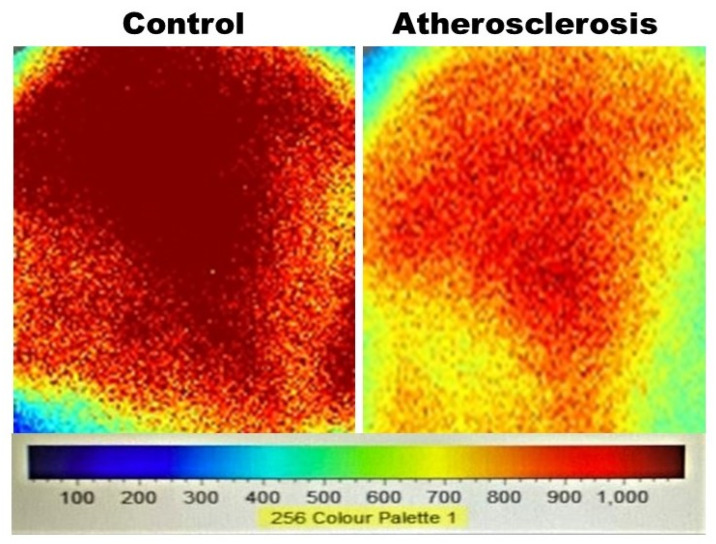
This figure shows samples of bladder microcirculatory blood perfusion measurement with the Speckle Contrast Imaging system in an animal with iliac artery atherosclerosis in comparison with blood perfusion measurement in a sham control animal. Statistical analysis of the blood flow data revealed that the average bladder blood flow of 770 ± 120.31 perfusion units in animals with arterial atherosclerosis was significantly lower in comparison with 1127 ± 219.01 perfusion units in sham controls (*p* = 0.013), suggesting bladder ischemia in animals with arterial atherosclerosis. Analyzed blood flow values are presented as mean ± standard deviation.

Measurements of bladder microcirculatory blood flow with the Moor FLPI-2 Laser Speckle Contrast Imaging system provided full-field laser recording and detected mean microcirculatory blood flow up to a tissue depth of 3 mm. Using this measurement system, we compared the bladder blood perfusion of rats with iliac artery atherosclerosis versus sham control rats. Statistical analysis of blood flow images suggested significant bladder ischemia in animals with arterial atherosclerosis. Ischemia was characterized by a statistically significant decrease in bladder blood perfusion in animals with atherosclerotic occlusive disease in comparison with sham controls, as shown in Figure 1. 

### 3.2. Differential Protein Modifications in Bladder Ischemia

In-depth analysis of a previously reported proteomic dataset [31] suggested that ischemia compromises the bladder proteome. These findings, along with our previous reports [31,32], imply that ischemia may play a key role in differential expression and post-translational modifications of proteins. The LC-MS/MS analysis of rat bladder tissues in our previous proteomic study showed that 172 proteins are upregulated and 527 proteins are downregulated in the ischemic bladder in comparison with control bladder samples (*p* ≤ 0.05) [32]. Proteomic analysis with consideration of at least a twofold increase or decrease revealed 97 up-regulated and 262 down-regulated proteins in the ischemic bladder in comparison with control samples [32].

Further analysis of the ischemic bladder proteome compared to controls revealed that 12 of the 23 amino acid variations were significantly dysregulated (R^2^ > 0.5, ratio > twofold, *p* < 0.05), suggesting potential ischemia-regulated protein modifications [31]. Interestingly, some of the amino acid variations provoked by ischemia that matched the molecular weights of the amino acid substitution did not corroborate the point mutations at the DNA and/or RNA levels, suggesting the production of non-coded amino acids (ncAAs) that might have resulted from post-translational modifications in the ischemic bladder. It is thought that ncAAs are produced in the process of amino acid substitutions or post-translational modifications of proteins. However, the precise nature of ncAAs and the underlying mechanisms regulating their formation remain elusive and warrant further investigation. Up-regulated and/or down-regulated ischemia-associated ncAA-containing proteins in bladder ischemia are summarized in Table 1 below.

**Figure 2:**
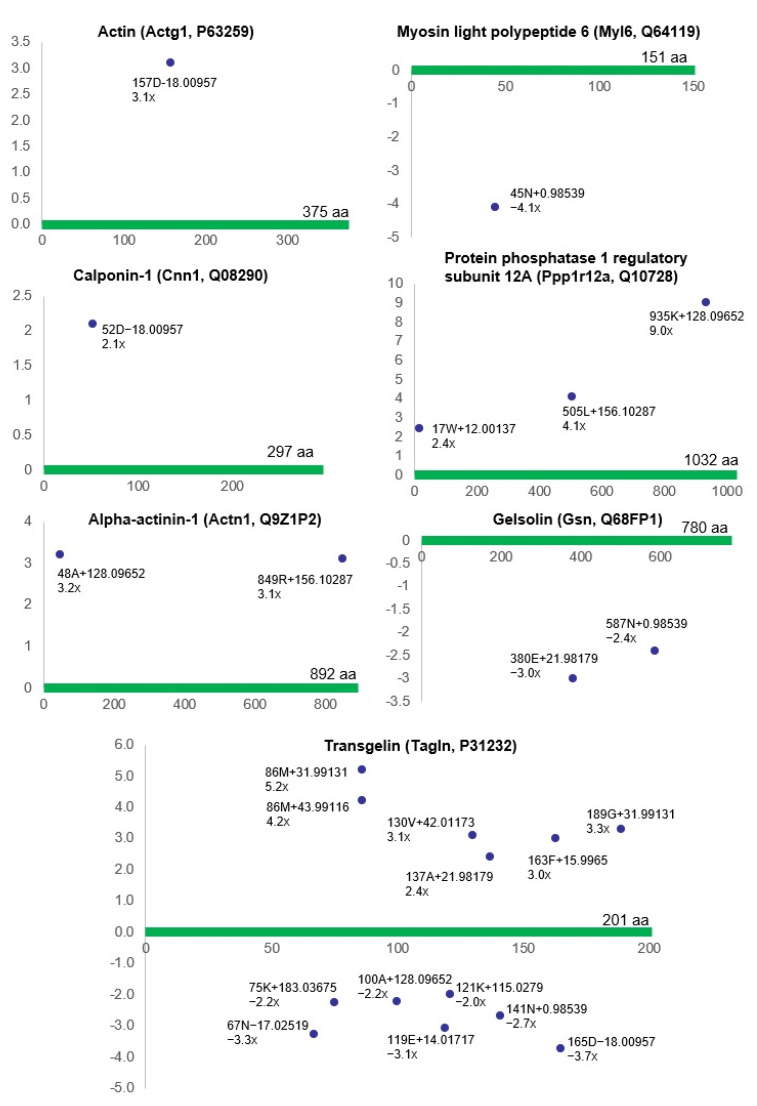
This figure shows the modified residues of contractile proteins and their associated proteins (R^2^ > 0.5, ratio > 2, *p* < 0.05). Each dot represents the ncAA of a specific protein. The annotation information for each dot is as follows: modified (protein) position, modified amino acid residue, delta mass, and ratio of modification. The horizontal axis represents the amino acid position of each protein; the vertical axis represents the ratio of modification (up or down). aa = amino acid.

**Figure 3:**
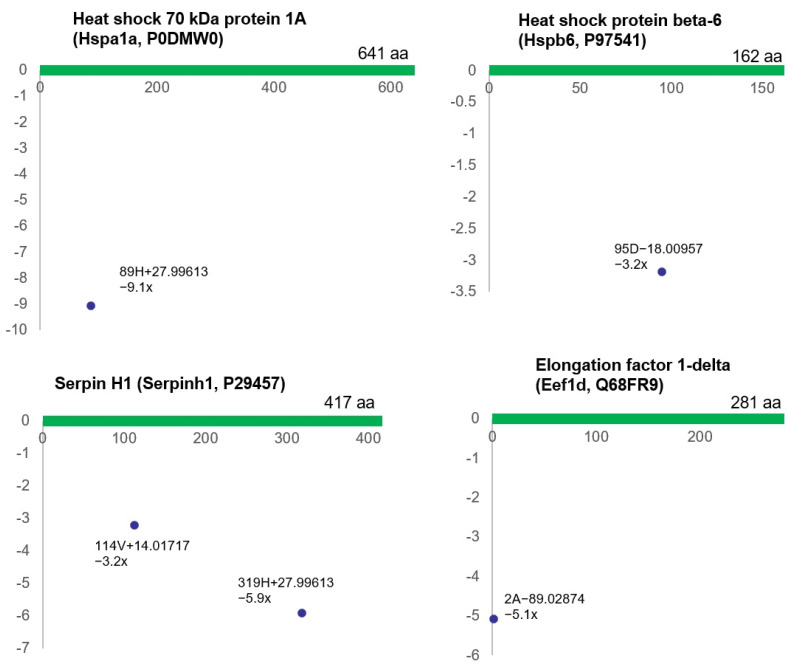
This figure shows the modified residues of stress response proteins (R^2^ > 0.5, ratio > 2, *p* < 0.05). Each dot represents the ncAA of each protein. The annotation information for each dot is as follows: modified (protein) position, modified amino acid residue, delta mass, and ratio of modification. The horizontal axis represents the amino acid position of each protein; the vertical axis represents the ratio of modification (up or down). aa = amino acid.

**Figure 4:**
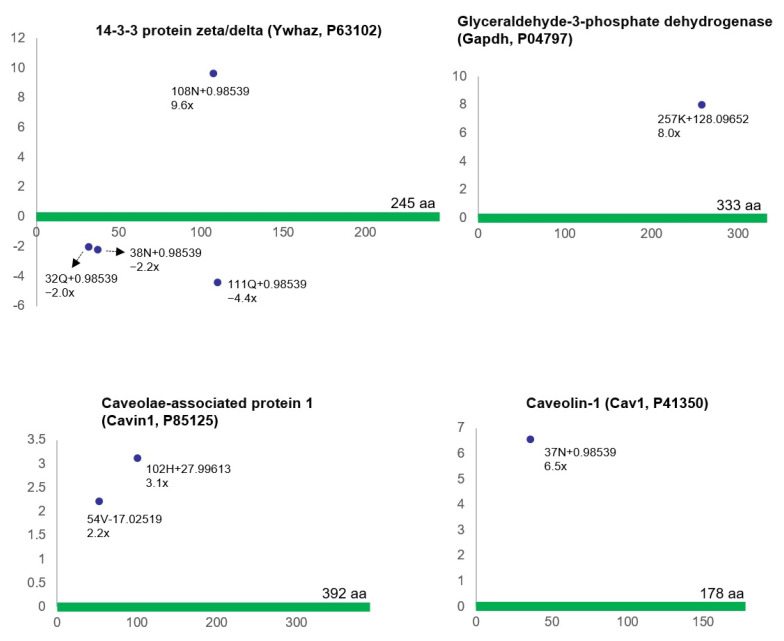
This figure shows the modified residues of cell signaling-related proteins (R^2^ > 0.5, ratio > 2, *p* < 0.05). Each dot represents the ncAA of each protein. The annotation information for each dot is as follows: modified (protein) position, modified amino acid residue, delta mass, and ratio of modification. The horizontal axis represents the amino acid position of each protein; the vertical axis represents the ratio of modification (up or down). aa = amino acid.

Proteomic analysis in our present study suggested that three groups of proteins in the bladder proteome, including contractile proteins and their associated proteins, stress response proteins, and cell signaling-related proteins, are conspicuously impacted by ischemia, as shown in Table 1 and Figure 2, Figure 3 and Figure 4. Widespread differential (post-translational) modifications were detected in the contractile proteins and their associated proteins in the ischemic bladder smooth muscle. This included actin (*Actg1*, P63259), myosin light polypeptide 6 (*Myl6*, Q64119), calponin-1 (*Cnn1*, Q08290), protein phosphatase 1 regulatory subunit 12A (*Ppp1r12a*, Q10728), alpha-actinin-1 (*Actn1*, Q9Z1P2), gelsolin (*Gsn*, Q68FP1), and transgelin (*Tagln*, P31232) (Figure 2). 

Stress response proteins were also markedly affected by bladder ischemia, showing differential (post-translational) modifications of the proteins, including heat shock 70 kDa protein 1A (*Hspa1a*, P0DMW0), heat shock protein beta-6 (*Hspb6*, P97541), serpin H1 (*Serpinh1*, P29457), and elongation factor 1-delta (*Eef1d*, Q68FR9) (Figure 3). 

Bladder ischemia also provoked differential (post-translational) modifications of cell signaling-related proteins, including 14-3-3 protein zeta/delta (*Ywhaz*, P63102), glyceraldehyde-3-phosphate dehydrogenase (*Gapdh*, P04797), caveolae-associated protein 1 (*Cavin1*, P85125), and caveolin-1 (*Cav1*, P41350) (Figure 4). 

## 4. Discussion

The role of ischemia in overactive bladder and lower urinary tract symptoms (LUTS) has been documented by clinical measurement of bladder blood flow, which revealed a close link between decreased bladder perfusion and LUTS in elderly men and women [6,7,8,9,10,11,12,13,14,15,16,17,18,19,20,21,22,23,24,25,26]. It was shown that the severity of LUTS closely correlates with the degree of bladder ischemia [6,7,8,9,10,11,12,13,14,15,16,17,18,19,20,21,22,23,24,25,26]. An epidemiological study involving 36,042 patients reported a significant correlation between LUTS and peripheral arterial occlusive disease [24]. Several other studies have shown that human bladder blood flow decreases with aging in both men and women, and decreased bladder blood flow significantly correlates with reduced bladder compliance and the development of LUTS [6,7,8,9,10,11,12,13,14,15,16,17,18,19,20,21,22,23,24,25,26]. Studies of animal models have revealed that ischemia compromises the bladder proteomic profiles and instigates a cascade of downstream signaling pathways with critical structural and functional consequences [37,38,39,40,41]. These observations, cumulatively, suggest that therapeutic strategies to improve bladder perfusion could prevent or reverse detrusor overactivity and LUTS in patients with pelvic ischemia. Downstream mechanisms mediating overactivity and structural damage in bladder ischemia appear to involve disruption of cellular energy homeostasis, cellular stress, and cell survival signaling [37,38,39,40,41]. It is thought that the upregulation of cellular stress response molecules by ischemia activates homeostatic mechanisms to signal nutrient deficiency, hypoxic stressors, and cell danger [42,43,44,45,46,47,48,49,50]. Our present study suggests differential (post-translational) modifications of contractile proteins and their associated proteins, cellular stress response proteins, and cell signaling-related proteins in bladder ischemia. The contractile proteins and their associated proteins modified by ischemia included actin (*Actg1*, P63259), myosin light polypeptide 6 (*Myl6*, Q64119), calponin-1 (*Cnn1*, Q08290), protein phosphatase 1 regulatory subunit 12A (*Ppp1r12a*, Q10728), alpha-actinin-1 (*Actn1*, Q9Z1P2), gelsolin (*Gsn*, Q68FP1), and transgelin (*Tagln*, P31232). The stress response proteins modified by ischemia included heat shock 70 kDa protein 1A (*Hspa1a*, P0DMW0), heat shock protein beta-6 (*Hspb6*, P97541), serpin H1 (*Serpinh1*, P29457), and elongation factor 1-delta (*Eef1d*, Q68FR9). The cell signaling-related proteins modified by ischemia included 14-3-3 protein zeta/delta (*Ywhaz*, P63102), glyceraldehyde-3-phosphate dehydrogenase (*Gapdh*, P04797), caveolae-associated protein 1 (*Cavin1*, P85125), and caveolin-1 (*Cav1*, P41350).

Post-translational modifications (PTMs) involve the loss of structural integrity of one or more amino acid residues resulting from the addition or removal of modifying groups on a translated protein for covalent processing modification [51,52,53,54,55]. Functional consequences of PTMs depend on the domain and motif that regulate cell function and changes that could provoke cellular structural damage, cell dysfunction, and the development of pathological conditions. The process of PTMs involves numerous episodes, including phosphorylation, acetylation, methylation, ubiquitination, glycosylation, and succinylation [51,52,53,54,55]. These processes play key roles in various aspects of cellular function and structural modifications [51,52,53,54,55]. For example, the intracellular concentration of sodium and potassium ions is regulated by the phosphorylation of the Na^+^/K^+^-ATPase pump on the cell membrane [52]. Acetylation may compromise protein conformation and alter interactions with substrates and cofactors [53]. Methylation of proteins regulates cell function by altering the original structural sequence of peptide chains, or could provoke dysfunction through the activation of downstream mechanistic pathways [54]. Ubiquitination can regulate cell function or mediate signaling pathways instigated by pathological conditions involving protein degradation or blockade of protein interactions [55].

It has been shown that ischemia may be a key player in provoking PTMs in a variety of cells [56,57,58,59,60,61,62,63]. The process of PTMs in ischemia appears to involve oxidative insult resulting from excessive production of free radicals in the ischemic cells [56,57,58,59,60,61,62]. The accumulation of free radicals instigates modification of the translated protein by inserting or detaching modified groups from the amino acid residues [56,57,58,59,60,61,62]. Oxidative insults mediated by free radicals in the ischemic tissues were shown to modify the fibrinogen molecule and provoke inflammatory responses [59]. DNA damage associated with histone PTMs was shown to play a key role in retinal neurovascular degeneration following ischemia/reperfusion injury [61]. Transient cerebral ischemia was shown to activate various protein PTMs involved in key neuronal functions and signaling pathways [62]. These observations suggest that the pathogenesis of chronic ischemia and ischemia/reperfusion injury is closely related to PTMs of proteins at functional domains [56,57,58,59,60,61,62,63]. It is thought that ischemia and oxidative stress compromise the physical and chemical properties of proteins that synchronize cell function in living organisms or elicit pathophysiological development in disease conditions [56,57,58,59,60,61,62,63]. However, certain limitations in protein sequencing technologies prevent the precise determination of global protein modifications throughout the proteome [63]. Therefore, it is difficult to precisely determine how many protein modifications are involved in tissue ischemia and how many underlying mechanisms are activated by PTMs under ischemic conditions. As each modification is likely to alter cellular structure and lead to functional consequences, systematic assessment of protein PTMs may be a convenient approach to examine molecular mechanisms and determine downstream pathways in the ischemic bladder.

Our previous LC-MS/MS-based proteomic approach detected 4277 proteins in the ischemic bladder and 4602 proteins in the control bladder [32]. Spectral count analysis revealed 172 up-regulated proteins and 527 down-regulated proteins in the ischemic bladders in comparison with controls [32]. Gene ontology analysis of the altered proteins in bladder ischemia based on their biological properties suggested the involvement of 66 ischemia-regulated proteins with a greater than five-fold change in functional categories regulating protein metabolic processes, proteolysis, and hydrolase activity [32]. Gene ontology analysis based on the molecular function of the 66 altered proteins implied their involvement in regulating peptidase and other enzymatic activities in bladder ischemia [32]. Canonical pathway analysis suggested a close link between altered proteins with a greater than two-fold change in bladder ischemia and several signaling pathways, including the ubiquitination pathway, the nuclear factor-erythroid 2-related factor 2 (NRF2)-mediated oxidative stress response pathway, and the extracellular-signal-regulated kinase (ERK)/mitogen-activated protein kinase (MAPK) pathway [32]. Network analysis revealed a link between altered proteins and cytoskeleton organization, cell death, and glucose metabolism disorders in bladder ischemia [32]. In another proteomic study, we found that ischemia provokes the differential formation of non-coded amino acids (ncAAs) in the bladder through mechanisms involving post-translational protein modifications and amino acid substitution [31]. Gene ontology analysis using Protein ANalysis THrough Evolutionary Relationships (PANTHER) revealed that the largest group of ischemia-associated ncAA-containing proteins in bladder ischemia relates to the cytoskeletal proteins [31]. Search Tool for Recurring Instances of Neighboring Genes (STRING) analysis of the protein-protein interaction network among the up-regulated ischemia-associated ncAA-containing proteins suggested that the protein-protein interaction networks were broadly grouped into three clusters: cytoskeleton-associated proteins, cell signaling-related proteins, and binding/transport-related proteins [31]. STRING analysis also suggested that interaction networks between the down-regulated ischemia-associated ncAA-containing proteins were broadly grouped into three clusters: cytoskeleton-associated proteins, metabolic enzymes, and molecular chaperones [31]. In our present study, a comprehensive analysis of the ischemia-associated ncAA-containing proteins revealed that three groups of proteins in the bladder proteome, including contractile proteins and their associated proteins, stress response proteins, and cell signaling-related proteins, are conspicuously impacted by ischemia. Widespread differential (post-translational) modifications were detected in the contractile proteins and their associated proteins in the ischemic bladder smooth muscle, including actin (*Actg1*, P63259), myosin light polypeptide 6 (*Myl6*, Q64119), calponin-1 (*Cnn1*, Q08290), protein phosphatase 1 regulatory subunit 12A (*Ppp1r12a*, Q10728), alpha-actinin-1 (*Actn1*, Q9Z1P2), gelsolin (*Gsn*, Q68FP1), and transgelin (*Tagln*, P31232). The actin cytoskeleton plays a key role in the configuration of subcellular structures and the preservation of cell function [64]. We have previously reported that bladder ischemia compromises actin cytoskeleton signaling and provokes aberrant smooth muscle contractile activity characterized by smooth muscle hypersensitivity to contractile stimuli [38,39,41]. Our findings are consistent with studies in the heart showing that oxidative modification of actin plays a key role in the pathophysiology of ischemic disorders [64,65,66]. It was shown that the oxidation of cysteines in actin interferes with the polymerization/elongation of G-actin and enhances F-actin fragility [67,68,69]. In cardiomyocytes, S-nitrosylation of α-actin promotes relaxation while impairing contraction [70]. These observations suggest that oxidative modification of actin may contribute to overactive smooth muscle contractions and detrusor overactivity in bladder ischemia. Ischemia-induced PTMs may compromise the dynamic properties of actin, provoke actin depolymerization, and sensitize actin to contractile stimuli.

Proteomic analysis of ischemia-associated ncAA-containing proteins also revealed that bladder ischemia elicits differential (post-translational) modifications of the proteins involved in cellular stress response, including heat shock 70 kDa protein 1A (*Hspa1a*, P0DMW0), heat shock protein beta-6 (*Hspb6*, P97541), serpin H1 (*Serpinh1*, P29457), and elongation factor 1-delta (*Eef1d*, Q68FR9). Such reactions of the stress response proteins may suggest cellular responses to ischemia under environmental changes with complex homeostatic disturbances in the ischemic cells that could compromise subcellular elements and provoke macromolecular damage. Our study suggests a close link between the interruption of nutrients and oxygen delivery to the ischemic cells, the accumulation of free radicals and metabolic waste, and differential (post-translational) modifications of the stress response proteins in bladder ischemia. The cell’s fate after exposure to stressful conditions such as ischemia depends on the severity and duration of ischemia and the cell’s defensive capacity to confine stress, eliciting molecular responses including PTMs [37,38,39]. Cellular stress sensors may play a key role in regulating molecular responses and protein modifications in the ischemic bladder [37,38,39]. 

In addition, proteomic analysis of the ischemia-associated ncAA-containing proteins revealed differential (post-translational) modifications of the cell signaling-related proteins, including 14-3-3 protein zeta/delta (*Ywhaz*, P63102), glyceraldehyde-3-phosphate dehydrogenase (*Gapdh*, P04797), caveolae-associated protein 1 (*Cavin1*, P85125), and caveolin-1 (*Cav1*, P41350). Cell signaling-related proteins regulate cellular responses to a diverse, multifaceted, and alternating mix of signals involved in protein expression, protein localization, protein endeavor, and protein–protein interactions [56,71,72,73]. These proteins are involved in critical aspects of signal transduction, modulating critical cellular responses to a nearly unlimited set of stimuli [56,71,72,73]. Our data suggest that the differential (post-translational) modifications of cell signaling-related proteins in the ischemic bladder may vary depending on the sensitivity, duration, and dynamics of the cellular stress response to ischemia. Signal-dependent modifications in the expression levels and PTMs of cell signaling-related proteins may play an important role in the regulation of downstream processes including protein degradation, localization, and protein-protein interactions [56,71,72,73]. The activity of cell signaling-related proteins is tightly regulated so that the products of the responses that they control are only elicited at the appropriate time when cells are stressed and signal danger [56,71,72,73]. It seems that some of the regulatory mechanisms involve protein expression or protein localization, but crucial functional consequences result from the regulation of protein activity by PTMs of the signaling molecule, intramolecular crosstalk between different domains within the protein, and/or activation of crosstalk mechanisms with other proteins [56,71,72,73]. A limitation of our proteomic analysis may relate to the sample size of 3 animals per group. However, LC-MS/MS analysis yielded an extensive proteomic dataset with a very large amount of data to be analyzed and interpreted. In LC-MS/MS analysis, each sample was run three times. We extracted proteins from each animal, ran SDS-PAGE, and divided them into 10 gel fragments. After sample preparations (in-gel digestions) from each 10 fragments, each sample of 10 was analyzed three times in LC-MS/MS. Therefore, each animal generated 30 sets of MS/MS data, allowing proper data interpretation with exploratory data analysis.

## 5. Conclusions

The role of bladder ischemia as a contributing factor in detrusor overactivity and LUTS has been documented by epidemiological studies and clinical measurements of bladder blood flow that suggest a close link between vascular risk factors, pelvic ischemia, and the development of lower urinary tract dysfunction. Ischemia appears to provoke substantial changes in the proteomic profiles of the bladder. The proteomic approach in our present study detected a widespread differential occurrence of ncAAs in bladder ischemia that may result from post-translational protein modifications and amino acid substitution mechanisms. These modifications may contribute to the dysregulation of downstream pathways, with important structural and functional consequences. The profiles of differential (post-translational) modifications and subsequent dysregulation of corresponding pathways in bladder ischemia may suggest a high sensitivity of the bladder to reduced blood perfusion and hypoxia. Differential (post-translational) modifications of proteins provoked by ischemia may play a vital role in increased smooth muscle contractions, structural damage, and the development of an overactive bladder. Proteomic responses, including differential post-translational modifications of proteins in bladder ischemia, may provide the foundation for future research towards clinical translation to identify diagnostic targets and develop effective therapeutic strategies against bladder dysfunction and LUTS.

## Figures and Tables

**Table 1:** Proteins containing ischemia-associated ncAAs (R^2^ > 0.5, ratio > twofold, *p* < 0.05).

Proteins Containing Up-Regulated ncAAs				Proteins Containing Down-Regulated ncAAs			
Protein Name	Gene Name	Protein ID	^a^ Freq	Protein Name	Gene Name	Protein ID	^a^ Freq
Hemoglobin subunit beta-1	*Hbb*	P02091	5	ATP synthase subunit beta, mitochondrial	*Atp5b*	P10719	2
14-3-3 protein zeta/delta	*Ywhaz*	P63102	1	Albumin	*Alb*	P02770	7
Non-muscle caldesmon	*Cald1*	Q62736	2	40S ribosomal protein S4, X isoform	*Rps4x*	P62703	1
Glyceraldehyde-3-phosphate dehydrogenase	*Gapdh*	P04797	1	Heat shock 70 kDa protein 1A	*Hspa1a*	P0DMW0	1
Caveolin-1	*Cav1*	P41350	1	Serpin H1	*Serpinh1*	P29457	2
Alpha-actinin-1	*Actn1*	Q9Z1P2	2	Alpha-crystallin B chain	*Cryab*	P23928	2
Transgelin	*Tagln*	P31232	6	L-lactate dehydrogenase A chain	*Ldha*	P04642	2
Albumin	*Alb*	P02770	21	Creatine kinase B-type	*Ckb*	P07335	1
Polymerase I and transcript release factor	*Ptrf*	P85125	2	Collagen alpha-2(I) chain	*Col1a2*	P02466	2
Protein phosphatase 1 regulatory subunit 12A	*Ppp1r12a*	Q10728	3	Serine protease inhibitor A3K	*Serpina3k*	P05545	3
Major urinary protein	*—*	P02761	1	Oligoribonuclease, mitochondrial	*Rexo2*	Q5U1X1	1
Calmodulin-1	*Calm1*	P0DP29	2	ATP synthase subunit alpha, mitochondrial	*Atp5a1*	P15999	1
Histone H1.0	*H1f0*	P43278	1	Ig gamma-2B chain C region	*Igh-1a*	P20761	1
Glutathione S-transferase P	*Gstp1*	P04906	1	Elongation factor 1-delta	*Eef1d*	Q68FR9	1
Cytochrome b5	*Cyb5a*	P00173	1	Desmin	*Des*	P48675	4
Histone H1.4	*H1-4*	P15865	1	Sepiapterin reductase	*Spr*	P18297	1
Desmin	*Des*	P48675	5	Alpha-1-antiproteinase	*Serpina1*	P17475	4
Hemoglobin subunit alpha-1/2	*Hba1*	P01946	4	Myosin light polypeptide 6	*Myl6*	Q64119	1
T-kininogen 2	*—*	P08932	1	Lactoylglutathione lyase	*Glo1*	Q6P7Q4	1
Complement component C9	*C9*	Q62930	1	Selenium-binding protein 1	*Selenbp1*	Q8VIF7	1
Actin, cytoplasmic 2	*Actg1*	P63259	1	Ubiquitin carboxyl-terminal hydrolase isozyme L1	*Uchl1*	Q00981	1
Actin, aortic smooth muscle	*Acta2*	P62738	7	Myosin regulatory light polypeptide 9	*Myl9*	Q64122	3
Heat shock protein HSP 90-beta	*Hsp90ab1*	P34058	1	Gelsolin	*Gsn*	Q68FP1	2
Cysteine and glycine-rich protein 1	*Csrp1*	P47875	3	Phosphoglycerate mutase 1	*Pgam1*	P25113	1
Alpha-1-inhibitor 3	*A1i3*	P14046	4	Hemoglobin subunit beta-2	*—*	P11517	1
Calponin-1	*Cnn1*	Q08290	1	Heat shock protein beta-6	*Hspb6*	P97541	1
Tropomyosin beta chain	*Tpm2*	P58775	3	Cofilin-1	*Cfl1*	P45592	1
Alpha-enolase	*Eno1*	P04764	1	14-3-3 protein zeta/delta	*Ywhaz*	P63102	3
Tubulin alpha-1B chain	*Tuba1b*	Q6P9V9	1	Four and a half LIM domains protein 1	*Fhl1*	Q9WUH4	2
Prothymosin alpha	*Ptma*	P06302	1	Heat shock cognate 71 kDa protein	*Hspa8*	P63018	1
				Transgelin	*Tagln*	P31232	7
				Non-muscle caldesmon	*Cald1*	Q62736	1
				Heat shock protein HSP 90-alpha	*Hsp90aa1*	P82995	1

^a^ Freq (frequency) is the number of unique ncAAs for each protein.

## Data Availability

Our research data will be shared with the public through the National Library of Medicine (NML) PubMed Central website and presentations at scientific meetings.
